# Perioperative inflammatory index differences between pulmonary squamous cell carcinoma and adenocarcinoma and their prognostic implications

**DOI:** 10.3389/fonc.2025.1554699

**Published:** 2025-02-20

**Authors:** Yi Liu, Songping Cui, Jing Wang, Bin Hu, Shuo Chen

**Affiliations:** ^1^ Department of Thoracic Surgery, Beijing Institute of Respiratory Medicine and Beijing Chao-Yang Hospital, Capital Medical University, Beijing, China; ^2^ Mass General Cancer Center, Mass General Brigham, Harvard Medical School, Boston, MA, United States

**Keywords:** inflammatory indices, lung cancer, prognosis, venous thromboembolism, D-dimer

## Abstract

**Background:**

Perioperative inflammatory indices reflect systemic inflammatory responses and have been linked to cancer progression and prognosis. This study aims to explore the differences in perioperative inflammatory indices between lung squamous cell carcinoma (LSCC) and adenocarcinoma (LUAD) and their association with long-term outcomes.

**Methods:**

This study included 287 lung cancer patients who underwent curative resection between June 2016 and December 2017, comprising 61 cases of LSCC and 226 cases of LUAD. Perioperative baseline information and inflammatory cell counts were collected. Patients were followed up for a median duration of 76 months, during which disease-free survival (DFS) and overall survival (OS) were recorded. Cox regression analysis was used to evaluate the prognostic significance of inflammatory factor levels.

**Results:**

Significant differences were observed in white blood cell count and systemic inflammation response index (SIRI) between LSCC and LUAD (P < 0.05). Regression analysis identified age (OR=2.096, P=0.004), postoperative day 1 D-dimer level (OR=1.550, P<0.001), and Platelet-to-lymphocyte ratio (PLR) (OR=1.901, P=0.031) as independent risk factors for perioperative venous thromboembolism (VTE). Furthermore, open surgical approach (HR=2.437, P=0.016), tumor type (LSCC; HR=2.437, P=0.016), and PLR (HR=1.534, P=0.019) were independent risk factors for DFS.

**Conclusion:**

Inflammatory index is key predictors of perioperative VTE and DFS in lung cancer, emphasizing their critical role in prognosis.

## 1 Introduction

Lung cancer remains a significant global health concern, with non-small cell lung cancer (NSCLC) comprising 80%-85% of cases. In China, the incidence of lung cancer has been steadily increasing, maintaining its position as the leading cause of cancer-related mortality worldwide (1–4). Data from the 2020 Global Cancer Statistics by the International Agency for Research on Cancer indicate that China recorded approximately 820,000 new lung cancer cases and 715,000 deaths that year (5, 6). Among NSCLC subtypes, adenocarcinoma is the most prevalent, followed by lung squamous cell carcinoma (LSCC) (1, 7). Lung adenocarcinoma (LUAD) represents around 47% of cases in Western populations, while in China, it accounts for 55%-60% (8, 9).

Advancements in cancer screening methods and treatment strategies have led to an increase in the overall 5-year relative survival rate for all cancers, from 49% in the mid-1970s to 69% between 2014 and 2020. However, the 5-year relative survival rate for lung cancer has only improved from 12% to 27% during the same period (10). In China, the 5-year survival rate showed a slight increase from 2003 to 2015, but it remains below 20.0%, indicating a generally lower overall survival rate (11). The treatment and prognosis of LUAD and LSCC vary based on their respective types. Compared to LSCC, LUAD often shows greater sensitivity to mutation-targeted therapies. For both LUAD and LSCC, immunotherapy has demonstrated safety and efficacy alongside chemotherapy and targeted drugs (12). Immunotherapy offers new hope and treatment options for patients with chemotherapy resistance, advanced tumors, or those lacking driver gene mutations (13–15). The continuous development of immune biomarkers and immune-related gene signatures will enhance the ability to predict the efficacy of immunotherapy (16, 17).

In recent years, numerous studies have unveiled the intricate interplay between tumors and inflammation (18–20). Research has shown that tumorigenesis is often accompanied by genetic alterations, which activate inflammation-related mechanisms and shape the inflammatory microenvironment. Conversely, chronic inflammatory conditions have been found to significantly promote cancer progression (21). These findings highlight the bidirectional relationship between inflammation and tumor development. Biomarkers such as the monocyte-to-lymphocyte ratio (MLR), neutrophil-to-lymphocyte ratio (NLR), platelet-to-lymphocyte ratio (PLR), systemic immune-inflammation index (SII), and systemic inflammation response index (SIRI) reflect the host’s immune and inflammatory status and play a crucial role in guiding treatment decisions and prognostic evaluations in various malignancies (22–24). However, their differences and association with prognosis in LSCC and LUAD remain underexplored.

The aim of this study is to investigate the differences in perioperative inflammatory markers between LSCC and LUAD, and to explore the significance of inflammatory indices in short-term outcomes and long-term prognosis. The findings are expected to provide valuable insights for perioperative management and prognostic prediction of patients.

## 2 Materials and methods

### 2.1 Study population

We prospectively collected data on patients who underwent radical surgical resection for NSCLC in our hospital between June 2016 and December 2017.

The inclusion criteria are as follows: (1) age over 18 years; (2) pathologically confirmed NSCLC; (3) absence of distant metastasis; (4) no history of neoadjuvant therapy prior to surgery; (5) underwent radical lung cancer resection; (6) informed consent regarding the surgical procedure and research protocol was obtained from the patients and their families. Exclusion criteria are as follows: (1) concurrent acute pulmonary or other systemic infections; (2) preoperative diagnosis of venous thromboembolism; (3) incomplete follow-up data.

### 2.2 Clinical data collection and follow-up

We extracted relevant data from the hospital’s electronic medical record system, including patient demographics (e.g., age, sex), underlying conditions (e.g., hypertension, diabetes, coronary heart disease), laboratory parameters (e.g., neutrophil, lymphocyte, monocyte, and platelet counts, D-dimer), surgical details (e.g., approach, duration, bleeding volume), pathological findings (e.g., tumor type, TNM staging), and the presence of venous thromboembolism (VTE).

Venous blood samples were collected from all enrolled patients within 24 hours after surgery, followed by complete blood count analysis. Regarding biomarkers, NLR represents the ratio of neutrophil to lymphocyte counts, MLR is the ratio of monocyte to lymphocyte counts, PLR is the ratio of platelet to lymphocyte counts, SII is calculated as platelet count × neutrophil count/lymphocyte count, and SIRI is defined as monocyte count × neutrophil count/lymphocyte count.

All patients received prophylactic anticoagulation with low molecular weight heparin (LMWH) immediately after surgery, typically on the evening of the first postoperative day, provided there was no significant risk of bleeding, and continued until discharge. All patients underwent bilateral lower extremity color Doppler ultrasonography both preoperatively and postoperatively to assess for newly developed deep vein thrombosis (DVT) after surgery. Patients confirmed to have DVT or exhibiting significant symptoms of pulmonary embolism (PE) underwent computed tomography pulmonary angiography (CTPA) to confirm the presence of PE.

We conducted follow-ups with patients through telephone, outpatient visits, or inpatient observation until December 2023 or the patient’s death. Postoperative follow-ups were scheduled for the 1st and 3rd months, then every 3 months for the first two years, and every 6 months thereafter. The primary examinations included laboratory tests and chest CT scans. All patients were regularly followed up, with detailed records of tumor recurrence and survival status maintained, and disease-free survival (DFS) and overall survival (OS) were calculated until death or loss to follow-up.

### 2.3 Statistical analysis

Normally distributed measurement data are expressed as mean ± standard deviation, while non-normally distributed data are reported as median (interquartile range). Categorical data are presented as frequency (percentage). Continuous variables were analyzed using the Student’s t-test or Mann-Whitney U test for group comparisons, while categorical variables were evaluated using Pearson’s chi-square test or Fisher’s exact test. Logistic regression analysis was used to evaluate independent risk factors for postoperative VTE in NSCLC patients, and Cox regression models were applied to identify independent prognostic factors related to DFS and OS. Variables with a P-value less than 0.2 in univariate analysis were included in the multivariate analysis. Statistical significance was set at P<0.05, and data analysis was performed using SPSS version 26.0 and GraphPad Prism version 8.0.

## 3 Results

### 3.1 Basic perioperative information

This results demonstrated significant differences in clinical characteristics and surgical-related indicators between the LSCC and LUAD groups. LSCC patients were older (63.8 ± 8.8 years vs. 59.1 ± 9.1, P<0.001) and had a significantly higher proportion of males (95.1% vs. 36.3%, P<0.001). Regarding surgical indicators, LSCC patients were more likely to undergo open surgery (60.7% vs. 12.8%, P<0.001), had longer operation times (188.3 ± 51.9 vs. 172.4 ± 50.4, P=0.030). Additionally, a higher proportion of LSCC patients were in advanced stages (II+III, 63.9% vs. 29.2%, P<0.001), whereas early-stage patients (0+I) were more prevalent in the LUAD group (70.8% vs. 36.1%, P<0.001) (**Table 1**).

**Table 1:** Basic information of all patients in this study.

Variables	Total (n=287)	LSCC group (n=61)	LUAD group (n=226)	P
Age	60.1 ± 9.2	63.8 ± 8.8	59.1 ± 9.1	<0.001
Gender (male)	140 (48.8)	58 (95.1)	82 (36.3)	<0.001
Comorbidity				
Hypertension	80 (27.9)	16 (26.2)	64 (28.3)	0.747
Diabetes	30 (10.5)	5 (8.2)	25 (11.1)	0.516
Coronary heart disease	18 (6.3)	8 (13.1)	10 (4.4)	0.013
Smoking	108 (37.6)	53 (86.9)	55 (24.3)	<0.001
Surgical approach (Open)	66 (23.0)	37 (60.7)	29 (12.8)	<0.001
Duration of surgery	175.8 ± 51.0	188.3 ± 51.9	172.4 ± 50.4	0.030
Amount of bleeding	100(100)	200(200)	100(100)	0.012
TNM Stage				<0.001
0+I	182 (63.4)	22 (36.1)	160 (70.8)	
II+III	105 (36.6)	39 (63.9)	66 (29.2)	
Tumor differentiation				0.870
Poor	59 (20.6)	13 (21.3)	46 (20.4)	
Moderate and well	228 (79.4)	48 (78.7)	180 (79.6)	
Neutrophil (×10^9^/L)	11.2 ± 3.0	10.2 ± 3.0	11.5 ± 3.0	0.002
Lymphocyte (×10^9^/L)	1.0 ± 0.4	1.1 ± 0.4	1.0 ± 0.4	0.312
Monocyte (×10^9^/L)	0.4 ± 0.3	0.3 ± 0.2	0.4 ± 0.3	0.445
Platelet (×10^9^/L)	218.2 ± 60.3	222.3 ± 60.7	217.1 ± 60.3	0.551
VTE	37 (12.9)	10 (16.4)	27 (11.9)	0.358

In terms of laboratory parameters, LUAD patients showed significantly higher neutrophil counts compared to the LSCC group (11.5 ± 3.0 vs. 10.2 ± 3.0, P=0.002). However, there were no statistically significant differences between the two groups in lymphocyte, monocyte, or platelet counts (P>0.05). The incidence of VTE was higher in the LSCC group compared to the LUAD group (16.4% vs. 11.9%), but the difference was not statistically significant (P=0.358) (**Table 1**).

### 3.2 Analysis of inflammatory and hematological parameters between LSCC and LUAD groups

**Figure 1:**
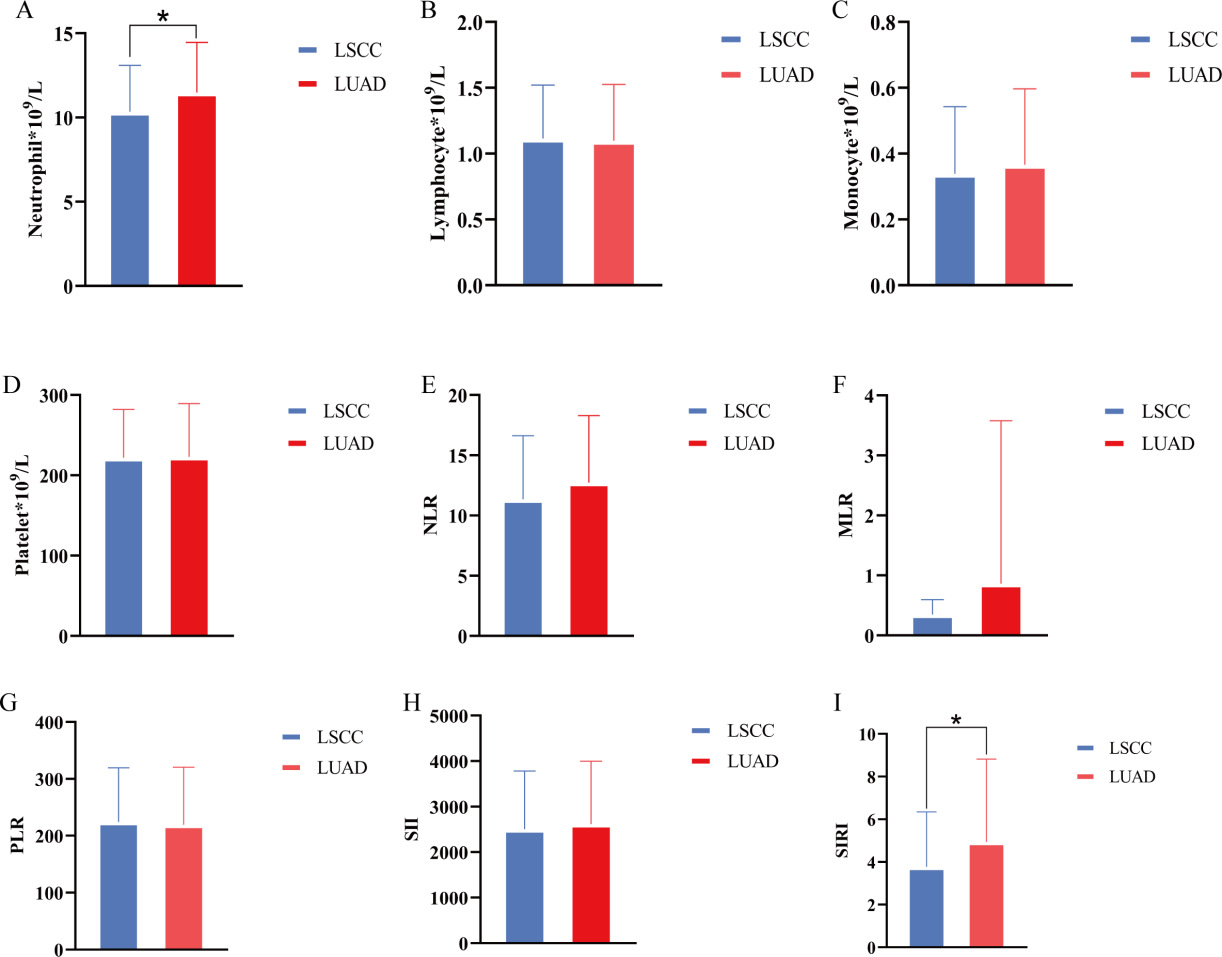
Differences in inflammatory cells and inflammatory indices between lung squamous cell carcinoma group and lung adenocarcinoma group. **(A–D)** Comparison of neutrophil, lymphocyte, monocyte, and platelet counts between groups; **(E–I)** NLR, MLR, PLR, SII, SIRI were compared between groups, respectively. *, P<0.05.

We conducted a comparative analysis between the LSCC and LUAD groups. The results showed that there were partial differences in inflammatory and hematological parameters between the two groups. The neutrophil count in the LUAD group was significantly higher than that in the LSCC group (**Figure 1A**, P<0.05), and the SIRI was also significantly elevated in the LUAD group (**Figure 1I**, P<0.05). However, no statistically significant differences were observed between the two groups in lymphocyte count, monocyte count, platelet count, NLR, MLR, PLR, or SII (P>0.05) (**Figure 1**). These findings suggest that the LUAD group may exhibit a more pronounced inflammatory state compared to the LSCC group, particularly reflected in the elevated neutrophil count and SIRI.

### 3.3 Analysis of risk factors for PVT

Logistic regression analysis revealed that several clinicopathological variables were significantly associated with postoperative VTE. In the multivariable analysis, age (OR: 2.096, 95% CI: 1.262-3.479, P=0.004), elevated D1 D-dimer levels (OR: 1.550, 95% CI: 1.228-1.958, P<0.001), and PLR (OR: 1.901, 95% CI: 1.059-3.412, P=0.031) were identified as independent risk factors for postoperative VTE. While open surgical approaches, prolonged operative duration, and intraoperative bleeding were significant in the univariable analysis, their associations were not retained in the multivariable model (**Table 2**).

**Table 2:** Logistic regression analysis of the effect of clinicopathological variables on postoperative VTE in patients.

Variables	Univariable Logistic regression		Multivariable Logistic regression	
	OR (95% CI)	P	OR (95% CI)	P
Age	1.074 (1.031-1.120)	0.001	2.096 (1.262-3.479)	0.004
Gender (male)	1.292 (0.644-2.591)	0.471		
Comorbidity				
Hypertension	1.110 (0.520-2.367)	0.787		
Diabetes	0.212 (0.028-1.603)	0.133	0.335 (0.035-3.186)	0.341
Coronary heart disease	1.382 (0.380-5.025)	0.623		
Smoking	0.770 (0.370-1.604)	0.485		
Surgical approach (Open)	3.048 (1.483-6.264)	0.002	1.040 (0.323-3.349)	0.947
Duration of surgery	2.018 (1.371-2.970)	<0.001	1.737 (0.941-3.206)	0.077
Amount of bleeding	1.157 (1.038-1.289)	0.008	1.129 (0.918-1.389)	0.251
TNM Stage (II+III)	1.565 (0.780-3.141)	0.208		
Tumor type (LSCC)	0.692 (0.315-1.522)	0.360		
Tumor differentiation (Poor)	1.126 (0.468-2.707)	0.792		
D1 D-dimer	1.597 (1.307-1.953)	<0.001	1.550 (1.228-1.958)	<0.001
NLR	1.036 (1.001-1.073)	0.044	1.029 (0.929-1.140)	0.579
PLR	1.003 (1.001-1.005)	0.015	1.901 (1.059-3.412)	0.031
MLR	1.296 (0.439-3.827)	0.639		
SII	1.156 (1.005-1.331)	0.043	0.709 (0.390-1.289)	0.260
SIRI	1.012 (0.926-1.107)	0.787		

### 3.4 Analysis of risk factors for DFS,OS

Cox regression analysis identified key factors influencing DFS and OS. For DFS, open surgical approach (HR: 2.437, 95% CI: 1.181-5.028, P=0.016) and PLR (HR: 1.534, 95% CI: 1.072-2.196, P=0.019) were independent predictors in the multivariable model (**Table 3**). For OS, advanced TNM stage (HR: 6.991, 95% CI: 3.123-15.653, P<0.001) was the strongest independent risk factor. These results highlight the prognostic value of tumor staging, inflammatory markers, and surgical factors in patient outcomes (**Table 4**).

**Table 3:** Cox regression analysis explored the impact of clinicopathological variables on disease-free survival.

Variables	Univariable Cox regression		Multivariable Cox regression	
	HR (95% CI)	P	HR (95% CI)	P
Age	1.127 (0.893-1.421)	0.314		
Gender (male)	0.678 (0.438-1.048)	0.080	0.476 (0.219-1.034)	0.061
Comorbidity				
Hypertension	1.234 (0.750-2.030)	0.407		
Diabetes	1.286 (0.583-2.838)	0.533		
Coronary heart disease	1.224 (0.592-2.532)	0.586		
Smoking	1.436 (0.912-2.259)	0.118	1.136 (0.507-2.547)	0.757
Surgical approach (Open)	1.634 (1.015-2.631)	0.043	2.437 (1.181-5.028)	0.016
Duration of surgery	1.055 (0.835-1.333)	0.653		
Amount of bleeding	1.019 (0.941-1.104)	0.642		
TNM Stage (II+III)	1.740 (1.054-2.872)	0.030	1.466 (0.728-2.955)	0.284
Tumor type (LSCC)	1.491 (0.830-2.677)	0.181	2.437 (1.181-5.028)	0.016
Tumor differentiation (Poor)	1.537 (0.980-2.410)	0.061	0.977 (0.515-1.856)	0.977
D1 D-dimer	1.073 (0.974-1.183)	0.153	1.092 (0.979-1.218)	0.113
NLR	0.959 (0.900-1.022)	0.197	0.952 (0.904-1.003	0.062
PLR	1.365 (0.896-2.078)	0.147	1.534 (1.072-2.196)	0.019
MLR	0.242 (0.027-2.165)	0.205		
SII	1.156 (0.758-1.763)	0.501		
SIRI	1.024 (0.867-1.210)	0.780		
VTE	1.951 (1.085-3.507)	0.026	1.165 (0.463-2.931)	0.746

**Table 4:** Cox regression analysis explored the effect of clinicopathologic variables on overall survival.

Variables	Univariable Cox regression		Multivariable Cox regression	
	HR (95% CI)	P	HR (95% CI)	P
Age	1.324 (1.019-1.721)	0.036	1.213 (0.881-1.670)	0.236
Gender (male)	0.754 (0.471-1.207)	0.240		
Comorbidity				
Hypertension	1.182 (0.701-1.994)	0.530		
Diabetes	0.794 (0.339-1.861)	0.596		
Coronary heart disease	2.565 (1.273-5.166)	0.008	1.117 (0.484-2.578)	0.795
Smoking	1.517 (0.947-2.432)	0.083	0.817 (0.458-1.457)	0.493
Surgical approach (Open)	3.455 (2.140-5.578)	<0.001	1.470 (0.736-2.935)	0.275
Duration of surgery	1.601 (1.236-2.074)	<0.001	1.282 (0.871-1.887)	0.207
Amount of bleeding	1.060 (1.004-1.119)	0.036	1.007 (0.907-1.119)	0.891
TNM Stage (II+III)	9.506 (5.355-16.876)	<0.001	6.991 (3.123-15.653)	<0.001
Tumor type (LSCC)	1.127 (0.627-2.025)	0.689		
Tumor differentiation (Poor)	3.605 (2.234-5.818)	<0.001	1.759 (0.975-3.176)	0.061
D1 D-dimer	1.176 (1.049-1.318)	0.005	1.065 (0.921-1.231)	0.394
NLR	1.021 (0.979-1.065)	0.334		
PLR	1.210 (1.039-1.410)	0.014	0.964 (0.639-1.452)	0.310
MLR	0.558 (0.231-1.351)	0.296		
SII	1.136 (1.041-1.239)	0.004	1.123 (0.874-1.444)	0.363
SIRI	0.995 (0.934-1.059)	0.868		
VTE	1.323 (0.694-2.519)	0.395		

## 4 Discussion

This study revealed significant differences in clinical characteristics and inflammatory indices between the LSCC and LUAD groups. LSCC patients were characterized by older age, a higher proportion of males, more advanced tumor stages, and a greater likelihood of undergoing open surgery. In contrast, LUAD patients exhibited a more pronounced inflammatory state, particularly reflected in elevated neutrophil counts and SIRI. Logistic and Cox regression analyses further identified age, D1 D-dimer levels, PLR, open surgical approach, and TNM stage as critical factors influencing postoperative complications and long-term prognosis, highlighting the key role of inflammation and tumor characteristics in prognostic evaluation.

In recent years, the significance of the tumor microenvironment (TME) has gained increasing recognition, with inflammatory cells and mediators playing critical roles in tumor initiation, progression, and immune regulation. Studies have shown that systemic inflammatory states not only influence the disease course in cancer patients but are also closely associated with postoperative survival and prognosis (25–27). The role of inflammatory cells and indices in assessing the prognosis and effectiveness of immunotherapy in NSCLC patients has attracted growing interest (27–31). In this study, we conducted a comparative analysis of inflammatory cells and indices between LSCC and LUAD patients. The results showed that postoperative neutrophil counts and SIRI were significantly higher in LUAD patients compared to LSCC patients. This difference may be associated with variations in clinical characteristics such as age, gender, and tumor staging between the two groups. The specific reasons and mechanisms underlying this phenomenon warrant further investigation in the future. Furthermore, in this study, the PLR was found to be significantly associated with postoperative VTE and DFS in lung cancer patients. Previous studies have shown that the PLR is an important prognostic indicator for long-term outcomes in patients with stage IV NSCLC and those receiving nivolumab therapy for NSCLC (26, 32–34).

Compared to neutrophils and lymphocytes, the correlation between platelets and tumors has received relatively less attention. The first report of platelet-related disorders in cancer was made by Armand Trousseau, who observed an increased risk of thrombotic events in cancer patients, a condition later named Trousseau syndrome (35). VTE, the second leading cause of death, is a significant complication in cancer patients and a common reason for hospitalization, substantially increasing cancer-related healthcare costs (36). Several cancers are associated with an elevated risk of VTE, including renal cancer, hepatocellular carcinoma, lung cancer, esophageal cancer, distal cholangiocarcinoma, pancreatic cancer, and gastric cancer, and it is correlated with poor long-term prognosis (37–42). The interaction between tumors and platelets is complex and plays a significant role in tumor progression and complications such as VTE. Studies have shown that tumor cells can attract and activate platelets, promoting the formation of fibrin clots and exacerbating thrombotic events (43). Additionally, platelets from cancer patients exhibit significant differences in mRNA expression profiles compared to those from healthy individuals, with tumor-derived stimuli inducing alternative splicing of platelet mRNA (44). Additionally, relevant studies have shown that platelets can preferentially accumulate cytokines and growth factors secreted by tumors, increasing cytokine concentrations up to approximately 10,000 times compared to plasma (45). Recent studies have shown that tumors communicate with platelets through small extracellular vesicles (sEVs), which deliver cancer biomarkers in a CD63-dependent manner and activate platelets, ultimately leading to thrombosis (46).

This study also has some limitations. First, this study was conducted at a single center, which may limit the generalizability of its findings to broader populations and diverse healthcare settings. Second, although key inflammatory indices such as PLR and SIRI were analyzed, other potentially important biomarkers, such as cytokines, were not included, which could contribute to a more comprehensive understanding of inflammatory processes and their prognostic significance. Additionally, while the study collected extensive clinical data, subtle differences in perioperative management and postoperative care may act as uncontrolled confounding factors, potentially affecting the interpretation of results. Finally, although the study identified significant associations between inflammatory indices and prognosis, it did not delve into the underlying biological mechanisms of these relationships. Future research should expand the sample size, conduct multi-center validation, and incorporate a broader range of biomarkers and mechanistic studies to enhance the generalizability and clinical applicability of the findings.

## 5 Conclusion

This study highlights the critical role of inflammatory indices, such as PLR and SIRI, in predicting perioperative VTE and DFS in lung cancer patients. Significant differences in inflammatory profiles between LSCC and LUAD underscore the importance of individualized approaches to patient management. By integrating inflammatory markers into clinical decision-making, this research provides valuable insights into risk stratification and prognosis, paving the way for more tailored therapeutic strategies in lung cancer care.

## Data Availability

The original contributions presented in the study are included in the article/supplementary material. Further inquiries can be directed to the corresponding authors.
